# Supplementary material for: Learning curve and surgical outcome of robotic assisted colorectal surgery with ERAS program
Source: Sci Rep. 2022 Nov 29;12:20566. doi: 10.1038/s41598-022-24665-w (PMC9709162; doi:10.1038/s41598-022-24665-w)
Supplement: Supplementary file 2 — Supplementary Information 2. [file 41598_2022_24665_MOESM2_ESM.zip › RAL Raw data-Ñ[▒K/087/Patho_ñw╝╨░O▒Kñσ.pdf]

臺中榮民總醫院 臺中市臺灣大道四段1650號 病理部活體組織切片 臨床醫學實驗及病理診斷中心 檢驗(查)報告

[redacted]

Pathologic diagnosis:
1. Descending colon, robotic left hemicolectomy --- Status post polypectomy with ulcer and granulation tissue. No residual lesion seen. The bilateral cut ends and mesocolic lymph nodes (0/9) are unremarkable.
2. Omentum, resection --- Fat necrosis.

Gross description:
The specimen consists of 1) a segment of colon, 10.5 cm in length and 5 cm in proximal cut end circumference, 4 cm in distal circumference, and mesocolic soft tissue. A scar-like lesion, 0.5x0.5 cm, labeled with surgical clip and surgical tie, is noted 4 cm to the distal cut end. On cross section, there is no tumor found grossly. The both cut ends are free of tumor grossly. 2) a piece of yellow soft tissue, 15.6x11.5x2.1 cm, labeled as "omentum". Representative part for section: A) proximal cut end B) distal cut end C-F) lesion site G) radial margin H-J) specimen 2 K-) lymph nodes. Blue ink indicated serosa; yellow ink indicated radial margin.

Comment:
Previous slides of colon polyp (S1016735) has been reviewed.
#T-59300_2 #M-45020_2 0 1105 00SP00
#T-C4560_2 #M-09183_2 0 1105 000000

已電子簽章

敬告：本單僅供醫師參考使用，不具任何申請效力！！
請病患妥為保管，若不慎遺失需自負個人資料外洩責任！
